# From genes to policy: mission-oriented governance of plant-breeding research and technologies

**DOI:** 10.3389/fpls.2023.1235175

**Published:** 2023-09-04

**Authors:** Maria Gerullis, Roland Pieruschka, Sven Fahrner, Lorenz Hartl, Ulrich Schurr, Thomas Heckelei

**Affiliations:** ^1^ Dyson School of Applied Economics and Management, Cornell University, Ithaca, NY, United States; ^2^ Institute for Food and Resource Economics, University of Bonn, Bonn, Germany; ^3^ Plant Sciences, Institute of Bio- and Geosciences 2, Jülich Research Centre, Jülich, Germany; ^4^ Wheat and Oat Breeding Research, Institute for Crop Science and Plant Breeding, Bavarian State Research Center for Agriculture, Freising, Germany

**Keywords:** research policy, governance, sustainability goals, plant phenotyping, automated phenotyping technologies

## Abstract

Mission-oriented governance of research focuses on inspirational, yet attainable goals and targets the sustainable development goals through innovation pathways. We disentangle its implications for plant breeding research and thus impacting the sustainability transformation of agricultural systems, as it requires improved crop varieties and management practices. Speedy success in plant breeding is vital to lower the use of chemical fertilizers and pesticides, increase crop resilience to climate stresses and reduce postharvest losses. A key question is how this success may come about? So far plant breeding research has ignored wider social systems feedbacks, but governance also failed to deliver a set of systemic breeding goals providing directionality and organization to research policy of the same. To address these challenges, we propose a heuristic illustrating the core elements needed for governing plant breeding research: Genetics, Environment, Management and Social system (GxExMxS) are the core elements for defining directions for future breeding. We illustrate this based on historic cases in context of current developments in plant phenotyping technologies and derive implications for governing research infrastructures and breeding programs. As part of mission-oriented governance we deem long-term investments into human resources and experimental set-ups for agricultural systems necessary to ensure a symbiotic relationship for private and public breeding actors and recommend fostering collaboration between social and natural sciences for working towards transdisciplinary collaboration.

## 1 Introduction

With Horizon Europe there is a €95.5 billion program fostering mission-oriented research and innovation in Europe (Mazzucato, 2019), entailing a new approach to research and its governance aiming to achieving the sustainable development goals (SDGs; Mazzucato, 2018). Mission orientation calls for a changed role of state and public organizations. Public organizations are supposed to act entrepreneurial, meaning they need to actively set innovation pathways and create markets, instead of only intervening in failed markets (Mazzucato, 2013). This implies a change in governance of research centered around specific, inspirational, yet, attainable goals, called missions (Mazzucato, 2018). Similar to the Apollo mission, putting a man on the moon, mission-oriented governance in Europe sets out with missions on, for example, climate-resilient regions (DGRI (Directorate General Research and Innovation), 2020b), beating cancer (DGRI (Directorate General Research and Innovation), 2020c), or healthy soils (DGRI (Directorate General Research and Innovation), 2020a). Mission goals need to be supported and brought about aided by appropriately governed research and innovation activities. We call these new efforts of governance ‘mission-oriented governance’. The different mission goals are developed such that they prioritize those systemic transformations with the best leverage towards reaching the SDGs (Sachs et al., 2019).

Achieving SDGs, demands that systemic transformation occurs in (1) education, gender, and inequality; (2) health, well-being, and demography; (3) energy decarbonization and sustainable industry; (4) sustainable food, land, water, and oceans; (5) sustainable cities and communities; and (6) digital revolution for sustainable development (Sachs et al., 2019). The agricultural sector is touched by all of these transformations: Be it through land-use efficiency, developing more productive plants, reducing food waste, impacts of unequal supply of education in rural areas, or applications of biotechnology in medicine amongst many others. Mission-orientated governance aims to facilitate these transformations from current agricultural production into sustainable agricultural systems (Sachs et al., 2019).

Core to sustainable agriculture is plants with improved properties and management practices allowing circularity and decoupling negative impacts (Pretty, 2018; Sachs et al., 2019). Currently plant production and breeding focus on increased yields, which needs to be extended to include other sustainability aspects, such as lower use of chemical fertilizers and pesticides, crop resilience to climate stress, and reduced postharvest losses (Qaim, 2020). Hence, plant breeding needs to provide the scaffold for efficient use of resources like water, nutrients, and minimization of pollutants in plant production. Targeted improvements of plants through plant breeding, however, are bound by evolving social and technological systems in research accelerating plant breeding.

Our objective is to propose a governance heuristic illustrating core elements needed for governing plant breeding research, such that its mission-oriented governance can achieve overall sustainability goals. Genetics (G), environment (E), management (M), and social system feedbacks (S) influence plant breeding outcomes. Symbolized by GxExMxS (as governance heuristic) we motivate, that holistic and systemic considerations need enter the creation of mission-oriented policy targets for plant improvements.

Yet, mission-oriented governance of agriculture creates a tension between how economists traditionally give policy advice on research and innovations in agriculture – with the state as intervening in failing markets (Alston and Pardey, 1996) - and a kind of governance centering around actively creating pathways of innovation. Hence, policy advice on mission-oriented governance focuses on a) directionality, b) dynamic evaluation, c) organization, and d) risk-and-reward sharing amongst public and private actors (Mazzucato, 2016). Directionality addresses how one may pick concrete targets and evaluation measures of effectiveness, which are broad enough to not stymie bottom-up exploration, discovery, and learning of involved actors within breeding contexts. Organizational challenges are related to building research infrastructures (RIs) advancing plant breeding providing sufficient absorptive capacity and long-run patience for high-risk undertakings, yet remain agile and innovative from within. This entails tackling how one can foster risk-and-reward sharing amongst public and private actors when RIs promise overall benefits. We adopt this approach in the following for research policy advice on mission-oriented governance of new approaches and technologies for phenotyping.

Phenotyping is the current bottleneck in developing advanced quantitative approaches to breeding needed for successfully creating improved crops (Pieruschka and Schurr, 2019). Developing ways for non-invasive high-throughput phenotyping and quantitative analytics is necessary for developing these new processes and tools for creating sustainable plant attributes. The European research infrastructure on plant phenotyping (EMPHASIS), currently being implemented, provides services like access to plant phenotyping facilities, competencies and data. Since 2002 the European Strategy Forum on Research Infrastructures (ESFRI) put forward the establishment of RIs integrated across Europe. RIs are public organizations that are supposed to provide access and other services to physical and virtual infrastructures for researchers across the EU (e.g. experimental facilities, biological samples, scientific data) and integrate national towards pan-European and global efforts (ESFRI (European Strategy Forum on Research Infrastructures), 2021). The RIs can develop their pan-European strategies towards providing research services and adapting RIs’ governance such that SDGs can be met in long-term. Accordingly, mission-oriented governance of plant breeding research – private and public - is supposed to support and bring forward breakthroughs in plant breeding research, and the EMPHASIS RI will be a vital part in implementing this strategy.

In the following, we first introduce the ‘nuts and bolts of plant breeding (section 2.1), then introduce what we mean by sustainability for agricultural systems (section 2.2) and how plant breeding in the past promoted and failed in achieving these goals. We highlight historic cases illustrating how genetics (G), environment (E), management (M), and social system (S) influenced plant breeding outcomes in the past (section 2). Symbolized by GxExMxS we motivate, that holistic and systemic considerations need enter the creation of mission-oriented policy targets for plant improvements (section 3). Then we introduce new modes and technologies of phenotyping, which will change and accelerate plant breeding processes (section 4.1). We discuss related economic implications for variety development in governing individual breeding programs (section 5.1) and point at potential challenges and bottlenecks in reaching sustainability goals (section 5.2). We then illustrate what mission-orientation under the premise of sustainability means for the governance of RIs developing phenotyping technologies and potential threats to their effectiveness (section 5.3 and 5.4) before concluding.

## 2 From genes to institutions – history and governance of plant breeding towards sustainability

In the following, we first describe basic terms for plant breeding. Then we illustrate the role plant breeding plays for the sustainability of societies and how we use the term sustainability for this paper. We then illustrate with historic cases what role phenotyping played in plant breeding and how modern advances in phenotyping technologies evolved from past challenges in sustaining societies.

### 2.1 Key terms in plant breeding

Phenotyping is observing the appearance of a plant and evaluating its products (Fiorani and Schurr, 2013). It is vital for plant breeding being concerned with selecting amongst different candidates those variants of plants showing superior attributes, also called traits (Becker, 2011). Breeding processes usually aim at a dedicated breeding target, a combination of superior attributes. Breeding targets are for example improving yield, resistance to pathogens, or having a certain quality, such as baking qualities. All observable measures, as they appear in a plant, are termed phenotype. The phenotype, however, is connected to the genotype.

A genotype is the genetic material of an organism and hence carries the hereditary information recorded in the organism’s genome. Changes in the genotype lead to changes in the plant’s phenotype dynamically interacting with its environment (Pieruschka and Schurr, 2019). Breeders usually denote this relationship between genotype (G) and phenotype (P) by using the formula P=GxExM with E for environment and M for the management of the plant. In practice plant scientists measure phenotypic traits under different conditions of environment and management (ExM) and connect these insights to the genetic makeup of the plant (G) (Becker, 2011). Plant scientists are usually more interested in how functional properties (like photosynthesis, transpiration, nutrient uptake) or structural properties (like shoot and root architecture, leaf size) of the plant are affected by the environment.

When looking at the genetic setup, bringing about phenotypes, researchers usually discern traits into complex (quantitative) and simple (qualitative) ones (Acquaah, 2007). Flowering time is an example of a simple trait, determined only by a few genes. Whereas, nutrient uptake or yield signify complex genetic traits being spread out over multiple loci on the genome. Plant phenotyping is particularly important to quantify the diversity of phenotypic traits and understand in which social and ecological contexts which genetic setup translates to which phenotypes.

Yield exemplifies how the different actors in the breeding system all have different perceptions and understanding of complex traits. Basic research in biology and plant science contributes to improving yield, by looking at the multitude of plant physiological traits influencing yield. For example, scientists try to understand how photosynthesis works in C3 compared to C4 plants telling us the range of yield in- or decrease in crops reacting to increased levels in CO2 in the atmosphere (Kebeish et al., 2007). These insights serve as theoretic background for pre-breeders.

**Figure 1:**
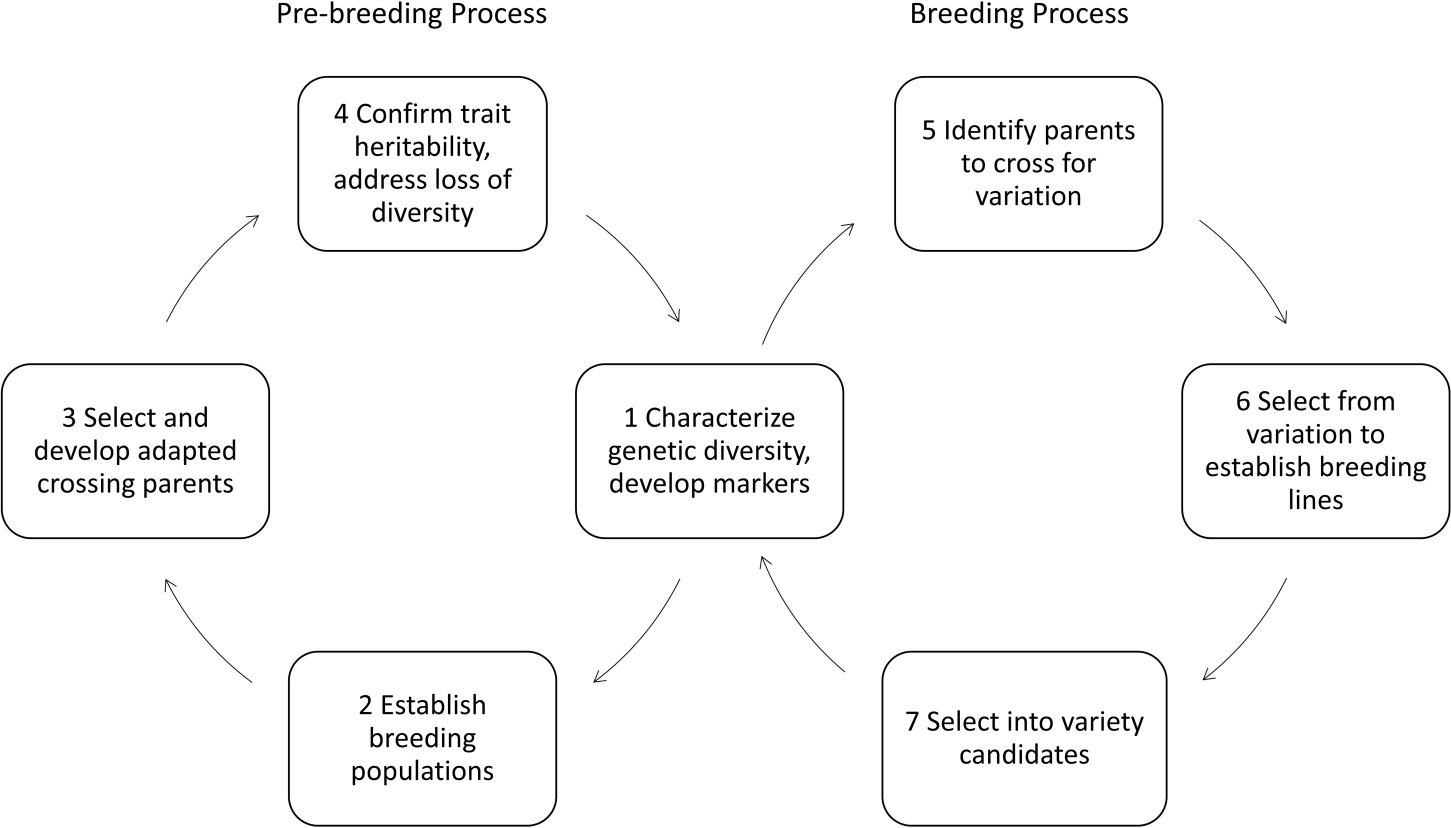
Pre-breeding and breeding processes adopted and extended from Watt et al. (2020).

Pre-breeders make some of these insights from basic research utilizable for breeding. They transfer knowledge about how single plants work to small populations of crops or introduce new genetic resources, for example from wild relatives to more adapted breeding material. They breed crops having advantageous new traits and bring about a yield level comparable to adapted varieties of a specific pedo-climatic region. Varieties are groups of homogenous, distinguishable plants of the same crop (Becker, 2011). Introducing new traits to a gene pool of already adapted varieties demands a lot of effort in pre-breeding (Gerullis et al., 2021). It is usually undertaken by partnerships between academia and industry (Moore, 2015; Gerullis et al., 2021). **Figure 1** shows the different steps of breeding and pre-breeding. Pre-breeders usually focus on selecting for those plants containing targeted traits into a better adapted genetic background with higher yields. This process usually takes years in practice (Gerullis et al., 2021), as complex traits need specific combinations of genes, being spread over the genome, whilst crossing-in new traits abates these efforts. Once new traits have entered an adapted gene pool, applied breeders can take these materials and cross them in with their breeding material (**Figure 1** box 1, 5 to 7). They create new varieties containing these new traits, aiming for best performance of all other important traits (higher average yields and qualities) by even better adapting these to a specific region. While applied breeders still include grain yield, seed weight, and resistances when they refer to yield, multipliers and farmers usually talk about yield in terms of tons per hectare.

Developing genetic markers for different traits necessitates characterizing genetic diversity (**Figure 1** box 1). Phenotyping provides here the necessary information to correlate genetic information with observations on how these genotypes perform under different environmental and management conditions, and how well different traits are inherited (**Figure 1** box 2 to 4). Phenotyping is basis for developing of molecular markers and genomics-based selection (Cooper et al., 2014). Automated systems in laboratory and field promise an increase in speed and precision in generating data and thereby accelerating pre-breeding and breeding processes.

Breeding outputs, namely varieties with improved properties, usually focus on improving yields, but also include other qualities, such as flowering colors, baking qualities, resistances to pests, nutrient content, or edibility. These are important to the remaining supply chain of agricultural and other plant-based products. It can take a decade or more to make a new variety of a crop. (Becker, 2011)

Plant breeding maintains and increases global productivity in agricultural products, see Laidig et al. (2017) for the contribution of breeding progress to yields and qualities in German winter wheat. Due to changes in the environment, breeders need to constantly adapt to changing conditions, and therefore maintaining the same yield level facing ever-changing pests can already be considered an improvement (see Olmstead and Rhode, 2008). Yet, as we are going to see in the following plants’ efficiency in resource use, their attributes in nutrient cycling and the systemic position cropping takes within the agricultural system determines how sustainable the overall system will be.

### 2.2 Sustainability by plant breeding?

In this section, we define what we mean by sustainable agricultural systems to clarify towards which goals we are heading, if we transform agriculture with mission-oriented governance. For this paper sustainability means that we can ensure the survival and thriving of humanity over an infinite time horizon. Doing so means living within the ecological boundaries of our planet (Rockström et al., 2009) while providing the social means to do so for all – as laid out by the SDGs (Raworth, 2017). Sustainable agricultural systems are social-ecological-technical systems (McGinnis and Ostrom, 2014) in which social, ecological, and technical processes produce food and fiber for the nourishment and fulfillment of the needs of humanity, while staying within the ecological boundaries of our planet (Rockström et al., 2017).

Sustainability of agricultural practices is in question if the current performance cannot be kept up in the long term. Some farming practices may lead to decreasing yields over shorter or longer periods and are as such intrinsically unsustainable. Whereas some are easily recognized in a short time (e.g. onion monoculture, Aragona and Orr, 2011) others involving soil erosion or accumulating salt may not be recognized by the individual farmer (see ancient Mesopotamian agriculture in Jacobsen and Adams, 1958; Gibson, 1974). Additionally, farming practices relying on resources that are not replenished as fast as they are being used are also non-sustainable. Phosphorus use for fertilizer or water use for irrigation are examples of it. The task of breeding in this context is to provide varieties that allow those agricultural systems avoiding such unsustainable practices.

Whether changes of traits by breeding are an ‘improvement’ depends on the boundaries of and the specific social-ecological context of the system considered. For example, if we breed plants for a cropping system with higher input of phosphorus, then this has implications not only for crop management but the whole supply chain of inputs related to it. Higher yields may directly impact the nutritional and income status of those growing the crops, yet phosphorus may need to be mined and economic and social conditions of those handling the resource on its way to the farm are impacted (Yiin et al., 2016; Nesme et al., 2018). If we, however, breed new traits into crops to use the phosphorous in the ground more effectively and have a lower phosphate extraction rate (van de Wiel et al., 2016), maybe some transportation of resources around the globe can be saved and additional extraction activities need not take place (Schipanski and Bennett, 2012). As we can see from this example, what to consider and how different changes in varieties affect sustainability depends on the context.

The overall direction of breeding goals for future cropping systems should consider context-specific resource-use efficiency, stability, or more generally, sustainability of farm and food system outcomes. Improving the ratio of relevant output to resources used such as land, water, energy, biodiversity, and other environmental pressures.

The adoption of high-yielding crops by farmers challenges plant breeders when it comes to incorporating other beneficial traits into their breeding programs. Farmers predominantly adopting crops with high potential yield (Lassoued and Smyth, 2023) favors varieties that promise just this at the expense of other characteristics like resistance to pathogens. Plant breeders face the dilemma of balancing expected yield enhancement with incorporating traits such as disease resistance, or other qualities (Gerullis et al., 2021). Incorporating new traits into breeding programs may initially lead to lower yields compared to ‘elite’ varieties, and yields have to be regained for example through backcrossing. This prolongs the breeding process and increases cost. Consequently, breeders must find innovative ways to address these challenges, striving to strike a balance between high yield potential and other traits, ensuring the long-term resilience and sustainability of agricultural systemsCrop traits shape crop production and we need to give plant breeding proper consideration in its role towards achieving the mission targets ahead. Hence, we point at new directions that may open up with new technologies and approaches to phenotyping in breeding research to navigate towards the SDGs more effectively. Yet, phenotyping technologies will not solve all challenges in bringing about sustainability and should not be treated as a panacea, as we elaborate in the following section.

### 2.3 Origins of phenotyping - or how to adapt genes to fit environmental conditions

Early forms of phenotyping were already employed in the rudimentary forms of plant breeding appearing when sedentism emerged. Having domesticated plants meant a vital step towards sustaining large-scale societies where agriculture serves using and bundling energy – sunlight – such that human societies can use it for better survival and thriving (Bätzing, 2020). As crops pose very specific demands on climate, soils, pathogens to survive, it is decisive to know which crop functions well in which environment to reliably secure nutrition and allow humans to pursue other purposes. Domesticating wild plant species into early crops through plain eye-sight, intuitive judgment and trial and error was thereby a form of mending the first plant-based biological technologies^1^ (Maisels, 1993; Becker, 2011).

Aggregating plants, through mass selection into landraces, can be counted as a process of cultural learning (Henrich and McElreath, 2003). Adapting plants, like the wild relatives of cereals, throughout domestication to the pedo-climatic conditions of the Fertile Crescent (Maisels, 1993; Brown et al., 2009), is a process of cumulative cultural evolution (Henrich and McElreath, 2003). The most important information of these early days of agriculture was enclosed in the genetic information of saved seed and could be propagated to the next generation by simple mass selection (Purugganan and Fuller, 2009). The accumulation of advantageous traits took several intermediate stages before certain crops were prominent over wider regions (Brown et al., 2009; Smith et al., 2019).

#### 2.3.1 The advent of scientific plant breeding

The advent of scientific plant breeding in the late 19th century stimulated more targeted breeding practices compared to the formerly used mass selection (Kloppenburg, 2004; Harwood, 2015). Breeders started to incorporate experimental designs (Mendel, 1866; Wieland, 2004). They generated scientific insights on-farm management and included the first mental models of the influences of genes on farm outputs (Brandl and Glenna, 2017). Breeders selected for more homogenous plant types (Kloppenburg, 2004; Wieland, 2004) and adopted more explicit and precise approaches to the underlying causalities assumed between plant physiological traits and farm outputs. They developed different forms of breeding and introduced the concepts of varieties, as uniform and stably performing groups of plants outperforming landraces in their yield by far (Becker, 2011). Meanwhile, crop genetic diversity reduced in richness (van de Wouw et al., 2010).

Approaching the management of crops with scientific methods emerged together with the different disciplines within the agricultural sciences (Wieland, 2004). They targeted higher yields by adding synthetic fertilizer and crop protection agents tested with experimental designs. Aiming for control of the natural environment in fields, by suppressing pathogens and weeds (Wieland, 2004). Coinciding, use of machinery increased, labor intensity decreased and productivity of western agricultural systems increased immensely (Olmstead and Rhode, 2008; Pardey et al., 2010). These scientific developments meant adding “M” to the basic formula of breeding, GxExM. This evolution in the agricultural sciences invoked the impression that the impact of the environment “E” was controllable by management practices (Wieland, 2004). Yet, pests constantly diminished the gains just realized by more targeted breeding (Olmstead and Rhode, 2008).

Discovering semi-dwarfed varieties, capable of producing comparatively higher yields, denoted a breakthrough in plant breeding (Pingali, 2012). Scientists, like Norman Borlaug, were capable of reversing a trait (long stems in cereals) brought about by natural selection in crops (Denison, 2012). Instead of further fueling individual competition between plants, dwarfing genes lead to plants putting their energy in higher grain yields and low stems, producing even greater outputs if fertilized (Denison, 2012). Developments in breeding went hand in hand with farm management advancements.

#### 2.3.2 Genotyping and biotechnology – answers to the pest problem?

Genotyping technologies invented in the 1980s allowed a deeper look into the genome. Breeders and pre-breeding scientists use these technologies to associate specific phenotypic traits, like a certain degree of susceptibility to a pathogen, with different mostly simple traits in the genome of crops (Eathington et al., 2007). Several genotyping techniques have been invented over the last two decades and have dramatically improved in terms of cost, speed, and accuracy for detecting correlations amongst gene loci and their phenotypic performance in different environments. While modern genotyping technologies permit to find those places in massive amounts of genetic data which bring about complex traits, limited data in phenotypes across different environments is available and hinders scientists to leverage their full potential. The data limitation in phenotypic information poses a bottleneck to advancing insight on how different genotypes perform in different environments (Fiorani and Schurr, 2013; Pieruschka and Schurr, 2019).

Explicitly taking genetic information into account for breeding opened up possibilities for modification. Pairing chemical mechanisms of pesticides with plant physiological traits, rooted in genetic modification (GM), was used to fight pests. Herbicide tolerance means that GM plants will survive a broad-spectrum herbicide where other weeds die (Kishore et al., 1992). Insect resistances for example induced through parts of Bacillus thuringiensis (Bt) genes lead to plants producing insecticidal proteins (Ranjekar et al., 2003). Yet, these alleged solutions to pressing pests are in vain from an evolutionary perspective, as the mechanisms employed to fend off pests are overcome by evolved resistances against these (Tabashnik, 2008; Carroll et al., 2014).

Denison et al. (2003) states that we merely enter an arms race between host plants and pests, but not resolving underlying problems. These cases of GMs^2^ represent low-hanging fruit in genetic modification and may even be misdirected in how they approach agricultural systems as a whole in face of natural selection. What seems successful at first produces no long-lasting improvements of agricultural systems. Natural selection caught up with human inventions, as these traits were used in big monocultural setups and pathogens had plenty of room for developing resistances to the employed chemical mechanisms (Denison, 2012; Søgaard Jørgensen et al., 2020). Consequently, the targeted plant protection starts to fail (Gassmann et al., 2011; see Tabashnik and Carrière, 2017 for an overview). In these cases, GMs add nothing new than what the application of pesticides and the co-evolving resistant weeds already did in conventional agriculture (Varah et al., 2020).

From an evolutionary perspective, Denison (2012) argues that humans are less likely to improve on those traits natural selection has been optimizing for millennia, but chances for improvement lie in redirecting natural selection. As plants face trade-offs in how they use their energy, some traits, stemming from increases in individual plant fitness, but unnecessary to human use, can be reversed for improvements towards human needs. Denison (2012) puts forward that for pests, there is no way of winning at the individual level, as plants and pathogens have been in these co-evolutionary cycles for long enough that natural selection developed plenty of strategies implemented in individual organisms to circumvent them. We can only hope to prolong a cycle in the arms race long enough to come up with new ideas of adaptation.

There is, however, a set of strategies aimed at changing agricultural practices on a collective level. When looking at pests from the perspective of an ecosystem, another set of possibilities opens up. Interrupting a pathogens propagation mechanism, for example by eradicating intermediate hosts (Olmstead and Rhode, 2002) as done with mulberries to eradicate black rust in wheat. These strategies alter a crop’s environment on a higher level. Strategies like these cannot be considered a mere change in management practices of an individual farmer, as they involve targeted collective action by farmers, extension services, and other interest groups on landscape level. The entailed social dilemmas, where incentive structures for individual costs and collective benefits diverge, can be quite complex, but have been achieved before with successful governance– see Olmstead and Rhode (2008) for more examples of historic accounts from the United States.

The principles and elements of agroecology as suggested by the FAO (FAO, 2018) target integration of social and ecological aspects for design and management of agricultural systems at a higher level (ecosystem level). Yet, Denison (2012) warns of false mimicry of whole ecosystems as it may lead to suboptimal outcomes compared to competitively tested systems. Competitive selection pressures of natural selection are not as effective on ecosystem level, as they are on individual (plant) level, as ecosystems usually do not compete for space against each other, opposed to individuals in ecosystems (Denison, 2012). Pest management is such an example, as any pest management strategy is counteracted by individual adaptations of pests. Yet, within an agroecosystem with homogenous conditions where a single strategy is being scaled up, pests are going to have an easier time for counteraction. Hence pest management strategies that employ diversity as a principle, for example refugia or crop mixtures with susceptible plants (Mallet and Porter, 1992; Tabashnik, 1994), need to be considered.

Independent of detailed strategies in governing agricultural practices, pest management is a good example to show, that aside of the fit between genetics, biological environment, and direct farm management practices, the social system and its governance on a higher-level needs consideration when developing targets for innovations in breeding. This will allow for achieving relevant individual and whole system-level outcomes (Carroll et al., 2014).

## 3 A governance heuristic for sustainability in plant breeding

Successful breeding demands very high R&D costs, which led to considerable concentration of firms in commercial seed markets (Deconinck, 2020) and the need for wise decision making in how and where public spending is directed. We can learn three things from the cases presented: One, not all traits are created with equal ease. We need to account for this in policy such that hard-to-get traits are developed by public monies, as private actors may be more likely to produce the low-hanging fruits. Two, the direction of genetic development is not open towards all possibilities. We need to account for what traits have been brought about by natural selection and where there is still room for development towards human needs. Three, the pest management examples highlighted above show that interactions of social-ecological dynamics lead to co-evolutionary cycles influencing cropping long-term. Short-term successes must not be overrated, as second-order effects on collective level may turn out to hamper overall systemic performance. We may need to find ways of slowing down arms races on wider systemic levels to have enough time for developing new adaptions.

While words like “social system” or “governance” may strike plant breeders and most crop scientists as a vague notion irrelevant to their work, we want to prevent exactly that and add an “S” for social system to the mental model of breeding and create a new heuristic for plant breeding governance:

GxExMxS

As explained, cropping outcomes rely on interaction of genetics (G), biological environment (E), directly applied management practices (M), and influences from the collective level implemented through governing the social system. Comparable to ecological environments higher-level social systems are complex in themselves. They are usually being structured by institutions (Ostrom, 2006) and entail all prescriptions bringing about individual-level behavioral patterns – usually subcategorized in rules, norms, and strategies, opposed to the laymen notion of an institution being an organization like a ministry. The management practices pointed out above are classical examples of strategies – describing what activities specific actors (e.g. farmers) perform. Norms and rules are usually brought about by different forms of governance systems, like cooperation organizations, lobby groups, or law-making bodies; they specify the conditions and sanctioning mechanisms under which individual strategies may or must (not) be enacted. Incentive alignment between individual strategies and the rules and norms brought about by the governance systems on all scales is key to structuring future breeding and farming systems.

We suggest the GxExMxS formula, see **Table 1**, as a governance heuristic to those people in policy advice and governance specific to plant breeding contexts. It should serve as a gentle reminder of not putting considerations of collective level activities in agricultural systems aside too quickly. For example, when EU project funds are being granted to researchers, funders should have some notion how activities scale up as this will influence the effectiveness of implementing innovations from plant breeding. Meanwhile, we want to encourage economists, who are traditionally good at considering markets and other institutions of governance, to more explicitly include notions of interactions between genetics and environment together with individual management and system-level outcomes.

**Table 1:** Governance heuristic for plant breeding research.

Definitions of GxExMxS	
G	“Genetics” – stands for changes on genetic/plant level.
E	“Environment” – denotes biotic (e.g. pathogens) and abiotic (e.g. soil and climate) environment of an agricultural system. It means those parts of the biophysical surroundings of locations where agricultural production or breeding takes place.
M	“Management” – means those activities undertaken by actors directly influencing plant growth in fields and controlled environments.
S	“Social” – implies the wider social system influencing management activities from the collective level but also co-evolving with the wider biophysical environment.

## 4 Phenotyping technologies

In the following section, we define and introduce automated phenotyping technologies and delineate underlying scarcities these breeding technologies may alleviate and point out bottlenecks they may bring about.

### 4.1 Overview of technologies in early research and development stages

Non-invasive high-throughput phenotyping technologies measure plant growth, structure, and composition with a specific precision in an automated manner, without destroying organs or canopy of the observed plant (Fiorani and Schurr, 2013). Being non-invasive, the new technologies enable observing plant traits without interrupting plant growth. Basic sensors and data processing may also be employed in farming, but plant breeding and pre-breeding pose different demands on these technologies, as they need to process smaller batches and have more heterogeneous tasks to fulfill (Watt et al., 2020). Sensor-based vision goes beyond the spectrum visible to humans’ eyes and even below ground, making it possible to observe new traits, only passively accounted for in breeding so far.

Researchers involved in breeding encounter scarcities in phenotypic data due to limited time and person-power. Precision and depth of data are usually an issue in collecting phenotypic data, depending on the physiological plant traits or farming outputs researchers are looking for. For example, daily images of the same plants throughout their growth period can be interlinked mathematically with genotypic information and daily climate data, for inferring how different genotypes may react to various weather conditions. Usually, multiple people need to score these attributes, by hand and eye inspection, while the source of data changes, once the person leaves the field, as plants continue growing. Main advantages of the new technologies are that one can see more, see faster, more precisely and there is no primary data loss.

For plant scientists there is a plethora of automated phenotyping technologies in different stages of development. **Table 2** presents an overview of the heterogeneity of phenotyping systems available for (pre)-breeding. All breeders must have some form of implicit or explicit notion about what and how inputs and efforts connect with their (pre)-breeding outputs, called mental models (Kieras and Bovair, 1984). Depending on their technical possibilities for inquiry, breeders use a variety of physical infrastructures for phenotyping: a) controlled conditions, like greenhouses or climate chambers, used alongside b) lean fields using minimal phenotyping equipment, like drones or robots or c) intensive fields using highly equipped for monitoring plants and environments. All physical infrastructure is complemented with d) information systems and e) modeling tools for processing sensor data.

**Table 2:** Overview of phenotyping technology types.

*Infrastructure category*	*Object of interest*	*Basic characteristics*	*Operational modes*	*Limitations/challenges*	*Examples*
*Mental Model*	Design of experiment	Denotes the functional and heuristic connections between breeding inputs and outputs	Present in all forms of breeding and pre-breeding practice: Implicit knowledge Explicit knowledge	Bound by computational capacity and information storage	• Breeder’s eye • Experimental designs and idea funnel
*Controlled Conditions*	Mostly single plants in pots (up to containers)	• Plant growth: plants are grown in growth chambers, greenhouse • Environment: well controlled environment • Capacity: 100-1000s plans per experiment • Experimental duration: days to weeks	Quantitative plant measurement using: Carrier system for plants PtS • Conveyor belts • Robotic systems Carrier system for sensors StP • Gantry systems Sensor: • Optical sensors (visible light (RGB), near infrared, multispectral, hyperspectral, thermal, fluorescence imaging, tomographic systems)	Often only small to medium sized plants possible	• WIWAM xy; • GrowScreen-Rhizo-1 • Phenotron Lemnatec
*Intensive Field*	Canopies in plots	• Plant growth: micro-plots usually in natural soil • Environment: Environmental monitoring (Semi-controlled conditions) • Capacity: 100-1000s plots per experiment • Experimental duration: Usually a growth season	Quantitative plant measurements: Carrier systems for sensors: • Fixed (e.g. towers, gantry systems) • Ground based mobile (e.g. phenomobiles) • Airborne mobile (e.g. drones) Sensors: • Optical sensors (visible light (RGB), near infrared, multispectral, hyperspectral, thermal, fluorescence imaging,)	Heterogeneous environment	• Breed-FACE • Pheno3C • Field Scanalyzer Rothamsted
*Lean Field*	Canopies in plots	• Plant growth: micro-plots usually in natural soil • Environment: Basic environmental monitoring • Capacity: 100s – 1000s of microplots multiple field sites • Experimental duration: Usually one or more growth seasons	Quantitative plant measurements Sensor carrier: • Ground based mobile (e.g. phenomobiles) • Airborne mobile (e.g. drones) Sensors: • Optical sensors (visible light (RGB), near infrared, multispectral, hyperspectral, thermal, fluorescence imaging,)	Heterogeneous environment	• Projects with networks of field trials (DROPS)
*Modelling*	Plants in silico (= virtual representation of phenotypes under different conditions)	• Virtual tools: –integrated in phenotyping pipelines (experimental design, image analysis) –interfacing with phenotyping pipelines (develop, validate in silico models)	In silico plant modelling • Process based models (e.g. simulate growth) • Functional structural plant models (e.g. plant architecture and physiology) • Statistical models • Models in phenotyping pipelines (e.g. trait quantification, dissection)	Need for experimental data	• Collection of models: https://www.quantitative-plant.org/
*Phenotyping Information Systems*	Data (all kinds of data, images and outcome measures)	• methods and interfaces for interoperability of datasets • manage, share, reuse and visualize heterogeneous, high-throughput plant phenotyping data stemming from different sources	Local information systems • Data base as part of a physical infrastructure for storage, visualisation data etc. • Data integration and reusability standardisation (data models, metadata)	Implementation of standards	• Data standards: MIAPPE (https://www.miappe.org/) BrAPI (https://brapi.org/ )

Source: based on Morisse et al. (2018) for updated list of examples from EU Infrastructure for plant phenotyping – EMPHASIS - see https://emphasis.plant-phenotyping.eu/phenotyping-landscape/infrastructure-map.

### 4.2 Controlled environments and enhanced vision traits

Controlled environments, in greenhouses and climate chambers, serve to investigate genetic variability in measured plant traits as a response to well-defined environmental conditions (**Table 2**). Researchers and breeders need to know the functional connection of how individual genes interact with each other as part of a plant and their environments. Most platforms can phenotype shoots throughout their growth period observing plant response when simulating biotic and/or abiotic environments, like temperature, water, nutrient availability, pathogens, etc. (example: PhenoTron in **Table 2**). Predominantly for controlled conditions the platform upon which the sensor system is mounted is fixed and plants are automatically moved to sensors creating observations (**Table 2**; Yang et al., 2020). Yet, there are also large installations where plants grow in fixed carriers and are being moved towards sensor systems – e.g., GrowScreen-Rhizo 1 (Nagel et al., 2012). Sensor systems are usually defined by noninvasive imaging measuring time series of dynamic processes such as plant growth. Depending on the trait of interest an entire electromagnetic spectrum can be used for different modes of imaging (Fiorani et al., 2012) usually in fully or semi-automated systems. With the current state of the art, most installations can process plants only until a certain stage in their growth – or only smaller crops and some platforms only permit to scan single plants, which decreases speed of inspection (Fiorani and Schurr, 2013).

Some platforms are capable of phenotyping roots below ground. While breeders have inspected above ground for thousands of years, to judge a plant’s quality, seeing it below ground opens up new possibilities to research. Now breeders can select for below-ground traits in a targeted manner. There are a few success stories demonstrating targeted selection of root traits (Watt et al., 2020). Being able to observe root setup without destroying them or their soil habitat over the growth period in an automated manner allows for data improving the speed in selection for root traits. This is essential for traits like water or nutrient use efficiency. These observations allow disentangling the role of root structures and their functional properties such as uptake of nutrients, biotic interactions within the rhizosphere (Watt et al., 2006). This brings about insights on genotype-to-phenotype relationships including those related to soil environments. (e.g. flood or drought stress, interactions between microorganisms and roots. We may be able to select entirely new trait types in applied breeding based on roots, where so far only shoot observations were used (Ober et al., 2021). So far, however, simultaneous measures of roots and shoots show that relationships between both are unpredictable, particularly for plant growth traits, like biomass (Nagel et al., 2015). Having more data available will likely give rise to disentangling these relationships.

Applying results from pre-breeding to practical breeding depends on how well genotypes predict intended outcomes, like yield, under field conditions. Yet, there are significant differences between controlled and field conditions in the target environments (Poorter et al., 2012) for example regarding light intensities or room for root expansion. It is impossible to fully simulate outdoor environmental conditions in experimental setups due to their complex dynamics (Kumar et al., 2015). Moreover, insight can usually only be gathered for smaller time spells in growth phases of a plant and rarely spans from seedling to harvest. Therefore, correlations between controlled environments and field conditions are generally fairly low (Kumar et al., 2015; Watt et al., 2020). Controlled environments allow predictions and heritability assessments of yield components where it may not be possible to assess those under field conditions. This allows directly developing insights for plants grown under controlled conditions, as needed for horticulture and vertical farming. However, phenotyping under field conditions is needed to see the performance of farming outcomes of different genotypes of crops farmed in large outdoor spaces.

### 4.3 Field environments and faster data generation

Field phenotyping serves testing plants – or rather their genotypes – under real environmental conditions. Testing plants in as many different environments of future potential relevance reveals the range of environments in which plant candidates perform well. This information can already be used for crop model simulations to scale up the variety’s ‘spatial reach’ (Grassini et al., 2015; Ersoz et al., 2020).

The range of technologies applicable for usage in the field is wide (Araus and Cairns, 2014; **Table 2**). Ensuring adaptability to differences in agricultural practice technologies range from rather low-tech field-bikes, with sensors mounted between two manually pushed wheels, robots looking like moving photobooths for cereals, or drones scanning fields. Most technology combinations of platforms and sensors currently tried out are mobile devices where the sensor is carried to the plants for imaging and can be distinguished by scanning single plants or multiple plots at a time. Some technologies are being developed for specific crops – like grapevines or sugar beet canopies – and therefore have limited flexibility in their technical setup (Schemenner and Tatikonda, 2005).

There are trade-offs involved at the technical level. Drones have the advantage of being faster at scanning a whole field than any human, yet resolution in their data is still limited (Burud et al., 2017). Drones do not need to navigate driving lanes or muddy fields nor do they compact soils. Yet, drones have trouble flying in adverse conditions with wind and rain (Chapman et al., 2014). Automated wheel-driven robots can easily produce high-resolution images of individual plants but still, take a lot longer than their human counterparts at scanning a whole field (Vijayarangan et al., 2018).

### 4.4 Socio-technological bottlenecks – data processing and management as the missing link

Scientists and breeders need to have actionable insights they can directly translate into their breeding practice. The knowledge about causal relationships between different factors and the phenotype is key to know what material to use next for breeding actual varieties. Scientists need to communicate these insights for breeders to use. Their experimental set-ups should enhance our understanding of relevant traits and their functional interactions of GxExMxS. Machine Learning is capable of compressing the high-dimensional ‘big data’ obtained and to produce predictions of phenotypical traits from genetic and environmental features (Minervini et al., 2015, Tsaftaris et al., 2016). To be able to employ machine learning, breeders need training or hire services/employees with the required new skills.

Another challenge is managing data for reusability. In pre-breeding, genotype-to-phenotype data in different environments is scarce, as a low number of candidate plants or seeds contain specific traits limiting repeated measurements. Meta-analyses could support robust insights on quantitative and qualitative traits (Watt et al., 2020). There are challenges involved in facilitating these studies: Data needs to be a) accessible, b) standardized/interoperable and c) worded in a common language (ontology), (d) findable (FAIR principle; Wilkinson et al., 2016) for describing what is being measured to make data comparable and re-usable across experiments. For meaningful comparison across different environmental contexts, pedo-climatic conditions, pathogen pressures, and other plant growth conditions need to be recorded systematically. Reusable data and replicable results are hard to gain under constantly changing environmental conditions (Massonnet et al., 2010). Ensuring FAIR data needs a collective effort by scientists and breeding practitioners complying with these principles. Several initiatives already exist aiming to harmonize experimental data from phenotyping, like the International Wheat Information System (http://www.wheatis.org/) or MIAPPE (https://www.miappe.org/).

## 5 The future of governing phenotyping technologies in plant breeding

High throughput phenotyping can contribute to sustainable intensification on different scales by shaping and accelerating crop improvements. Automation will influence individual breeding programs as they produce varieties with better traits than before. RIs provide the socio-technical environment and concrete demand-driven services to achieve this.

### 5.1 Implications for managing applied breeding programs

Breeding programs produce varieties for farmers to use. Private businesses try to recoup their research and development investments through sales of varieties or licenses for multiplication. Breeders are usually faced with the two-fold problem of creating variation of trait expressions in candidate variants and then selecting effectively and efficiently from the variation created for combinations leading to improved farm outputs. The number of varieties admitted for sale and income generated from sales or licenses can be seen as their current measure of success. Yet, these numbers need to be interpreted as relative to the inputs used by a breeding firm. (Gerullis et al., 2021).

Inputs - limiting factors to practical breeders’ operations - are nursery space, different locations for having a variety of environments available to test breeding lines under different conditions, genetic variation in their material, and skilled or unskilled person-power producing and evaluating the depth and breadth of data created through the mentioned factors of production. Breeders employ social strategies to work around the physical limits of their firm. Some breeders share and exchange information, nursery space in different locations, and material with their colleagues or co-produce new genetic traits with scientists in pre-breeding programs (Gerullis et al., 2021). Even small breeding programs can be quite successful as such (Brandl, 2018) if they manage their input to output ratio well and produce well-working varieties for different ecological niches.

Adopting high throughput phenotyping as an applied breeder leading a breeding program only makes sense if the technologies alleviate the resource scarcities mentioned and if they help outperform the return on investments necessary for the technologies of the breeding process currently in use. Those firms will be the most successful in employing the technologies that can leverage them for developing wider phenotypic variation and/or then employing the technologies for increased selection pressure, thereby accelerating the breeding process (Brandl, 2018).

Breeders’ mental models of the functional connection between crop physiological traits, genotypic information, and the phenotypic observations of varieties and farming outputs under different environments (biological and social) determine what breeders use in their breeding process. It is vital to know for a breeder how and when to inspect signs of a disease, for example, fusarium head blight in late growth stages shows a whitening of wheat ears, to look for resistance of the same (Champeil et al., 2004). They need to know how candidate variants perform under different disease pressures and then relate observable farm outcomes, like toxin levels in wheat harvests if they are susceptible to fusarium.

Sensors employed in high throughput phenotyping can enhance vision beyond plain eye-sight, opening up possibilities for completely new breeding input traits, so far ignored (Watt et al., 2020). Yet, for bringing about improved varieties, breeders’ mental models, depicting causal connections in terms of structure and processes of the plant system (Kieras and Bovair, 1984), are decisive. For example, if a higher-level goal for breeding is to boost plant productivity by introducing crop varieties paired with specific variants of mycorrhizae (Brito et al., 2021), then the tricky part for the pre-breeder is figuring out which plant physiological attributes an applied breeder needs to look out for to bring about improved farm outputs. Breeders need to know what patterns to look for in the images of root structures they gather and what these different patterns mean to formulate expectations of how crops work and how they can gain improvements. Additionally, opportunities arise where interactions of multiple factors come into play. For example, if different root structural patterns allow for a narrower planting on the same space, an increase in yield through interspecies cooperation (e.g. micro-organisms and plants) and variation in field arrangements (Grahmann et al., 2021) allows a push and pull pest control, then all three factors may be combined (Denison, 2012). Both examples need new ways of phenotyping and the integration of experimental meta-data into experimental set-ups of applied breeders.

Automation – once established – can bring about more comparable and precise measures of phenotypes across locations. In handicraft breeding, personnel have to hand-inspect and rate every variant plot for multiple time slots throughout growing seasons (Reynolds et al., 2019). There are differences in how individuals rate plots. Breeders usually compensate this by knowing their staffs’ style of judgment and triangulating the results for important diseases. Human staff will usually correct their ratings for environmental conditions. Some diseases may not be visible well if another disease already infected big parts of a plant or if only low disease pressure is present. Automated phenotyping and the corresponding image processing algorithms could, once machine learning models employed are trained to compensate for these problems, aid in inspecting and rating over multiple locations saving person power and time (Reynolds et al., 2019). Paired with decision support systems for breeders, which pre-process the data, there is potential for accelerating breeders’ work in this approach if robotics and data management systems can be maintained and adapted easily (Kuriakose et al., 2020). Yet, the additional data in terms of quality and quantity created needs to be processed, standardized and interoperable to work effectively (van Dijk et al., 2021).

### 5.2 Bottlenecks in breeding programs and opportunities for new service industries

Depending on their pre-existing socio-technological infrastructures, private breeders face different trade-offs when considering investing in automated phenotyping technologies. The cost and risks of investing in robotics-based phenotyping may be immense for a small breeding firm currently equipped with just the minimum technical setup for instance in wheat breeding – nursery fields, skilled and unskilled labor, and a rudimentary computer system where they store and manage data from plant inspections. The firm would need to invest, in the robot(s) itself, the highly skilled robotics personnel to implement, maintain and improve it and more personnel skilled in computer science for implementing, maintaining, and improving data and knowledge management and analysis (Reynolds et al., 2019). With shifting to new systems, firms run the risk that the new technology will cost more than it adds in value. Similar considerations struck breeding programs 35 years ago when they faced the integration of molecular genetics with plant physiology (Reece and Haribabu, 2007). Some breeding firms outsourced genotyping their seeds and a service industry appeared (Shkolnykova, 2020). This outsourcing generally worked better for some breeding programs, where the initially chosen interdisciplinary collaboration between molecular biologists and applied plant breeders was problematic (Reece, 2007; Reece and Haribabu, 2007). Today, smaller breeding programs use genotyping services to scan for specific markers, targeted genetic sequences, of intended breeding input traits and base their selection on the results. Using services for genetic markers in breeding accelerates breeding already.

Having more data from an automated phenotyping process will only increase value-added if the software for processing the new data types enables breeders to integrate their hypotheses into building new ideotypes, i.e. targeted ideal phenotypes. Software needs to be flexible enough to accommodate new insights when new traits are developed (Xu and Crouch, 2008). They need to contain graphical user interfaces, which allow for ample flexibility for the set-up of data processing through the breeder, without having to have a computer science degree (Galitz, 2007). It is important that breeders can individually fine-tune analyses and try out assumptions for different functional models between trait expressions and outcomes. Breeders need to be able to arrange their experimental designs for crossing and selecting according to their wishes. Breeders need to learn how to explicitly transform the “breeders’ eye” (Brandl, 2018) into heuristic computer models. Open question is whether breeders will actively engage in pre-breeding and try to develop different ideotypes, or go for merely applying what pre-breeding research serves to them as new ideotypes and use trial and error in application.

There is ample opportunity for specialized services to develop alongside new breeding technologies. Effortless usage and maintenance of robots and data infrastructures may be provided well by businesses, who arrange their activities around co-producing services for multiple breeders. We specifically say co-production, as these services demand a collective and dynamic learning process, based on research by universities and research institutes, then tailored to different localized social contexts, biological environments, and crops. In other sectors, like banking, the co-creation of technologies with heterogeneous small actors has brought about decentralized organizational structures and kept market concentration at bay (Hannan and McDowell, 1990). Considering how heterogeneous and locally adapted breeding needs to be to produce varieties fit for prevailing environmental conditions, long-run cooperative networks of firms may outperform single players in achieving this goal. Multiple firms may pool resources and share risks in developing software, data management services, and robots focusing on ease of use and flexibility for individual ideas and specific conditions. This way, a diversified approach of adopting the new technologies seems possible for breeders even if they currently possess low-tech infrastructure. As the case of German winter wheat shows (Brandl, 2018), cooperative breeding strategies have led to German wheat breeders outperforming the global competition over the last 100 years in terms of yields (Brandl et al., 2014). Going for co-production may in the long-term better hedge our bets for societal goals of sustainability overall, as we maintain flexibility and adaptiveness to localized conditions.

Accelerating the breeding process through increased selection pressure may bring about a trade-off over nursery space for short-term variety development and maintaining genetic resources in adapted breeding material (Gerullis et al., 2021). If automated phenotyping provides more precise predictions compared to current selection schemes, breeders will be quicker with selection decisions for dropping material. Meaning that breeders run the risk of dropping material earlier in the breeding process than before, possibly losing too much valuable variation in genotypes. Private incentives led to underinvestment in crop genetic resources in the past already in the USA (Day-Rubenstein et al., 2005). Hence, monitoring and evaluating *in-situ* genetic resources from breeders and their released varieties will be vital to ensure long-term functioning of seed production and needs to be developed alongside the new technologies. In the next section, we will go deeper into how public RIs can support these strategies and promote overall sustainability goals.

### 5.3 Implications for policy: threats and opportunities to effective research infrastructure governance

RIs provide resources and services for research communities conducting research and fostering innovation (ESFRI (European Strategy Forum on Research Infrastructures), 2020). From a mission-oriented perspective, a RI around plant phenotyping serves as an accelerator for developing agricultural systems adapted to existing or upcoming challenges. Developing these sustainable agricultural systems demands governance connecting scientists and all relevant stakeholders, providing physical and mental space to rigorously test different system configurations against each other. Principles of mission-oriented governance (Mazzucato and Li, 2021) necessitate a) defining overall but also intermediate goals, b) entertaining a widespread portfolio of project set-ups so that failures become acceptable, c) involving actors and investment across different scientific disciplines, private and public sectors, d) joined governance, yet, strategic division of labor among involved research sections with well-defined responsibilities for coordination and monitoring.

We put forward GxExMxS as rule of thumb for thinking about how efficiencies in land use, water, energy, ecological impacts due to changes in nitrogen, phosphorous, and carbon cycling are brought about, at different levels initiated and/or complemented by changes in traits of crops. Research programs under the Horizon Europe missions should integrate relevant stakeholders having expertise in different topics. RIs are supposed to function as an organization providing services such as access to facilities, data, resources and could function as an important element stimulating cross-disciplinary interaction and research towards common goals. With their cross-cutting capabilities to reach many different actor groups, RIs are key in shaping how governmental monies spill over to private industry (Mazzucato and Li, 2021). They can deploy mission-oriented organizations, to crowd-in private investment and use knowledge governance for public values, by putting in play conditionalities of public interest (Mazzucato, 2018; Mazzucato and Li, 2021).

Aside from immediate breeding outcomes, the performance on-farm and beyond must be considered as well, potentially already during pre-breeding. High-throughput installations need to be accessible to create high-quality, reusable data sets to yield reliable results for crop model predictions and integration into simulation models over larger spatial scales including different pedo-climatic zones. Basic research on crop improvement needs rigorous testing of different technical systems’ performance, necessitating flexibility in where and how different sensors are used. This demands modular installations, sensors, and platforms. Scientific testing and optimization must not stop until new system configurations outperform the best running systems in use on farms, to provide proper proof of concept ready for wider application. On the level of research, this includes from biological insights of symbiotic interactions amongst crops and other organisms to technical inventions developing enhanced vision with machines.

EU funding of RIs together with other fiscal incentive schemes for agricultural research aims at developing innovations for the Green New Deal (Mazzucato and McPherson, 2019) and achieving sustainable development goals with mission-oriented governance (Sachs et al., 2019). The goal is to crowd-in those individuals and organizations, who are willing to innovate for achieving these goals and co-creating new markets for and through sustainable innovations. RIs play a role as enabling scaffold in these overall European goals.

Yet, treating RIs merely as enabling organizations is not enough. Supporting the overall directionality of missions like healthier soils or adaptation to climate change (European Strategy Forum on Research Infrastructures, 2016; ESFRI (European Strategy Forum on Research Infrastructures), 2020) effectively not only requires the development of technological features, like steering software for robots, but collective learning across sectors and disciplines to achieve goals like the SDGs. As reaching the SDGs requires deep structural changes across all sectors of society (Sachs et al., 2019), they include social cooperation problems across multiple scales and amongst different stakeholders discussed in section 2.3. Leading actors in RIs may need to adopt institutional navigation as they pursue the SDG policy goals against a backdrop of complex, polycentric governance, where multiple decision-makers engage in different forms of organization to manage cooperation problems present in agriculture (Lubell and Morrison, 2021).

Facilitating a research environment with learning and high explorative capacity best fits for tackling the mission’s challenges (Mazzucato, 2015; Lubell and Morrison, 2021). High explorative capacity within these organizational structures may be achieved through a social environment where RI staff can welcome uncertainties and long-term competencies are developed (Mazzucato, 2015). Additionally, staff need to be proactive and entrepreneurial in their role of leading researchers and other actors using the infrastructure and its outputs (**Table 2** for examples).

**Figure 2:**
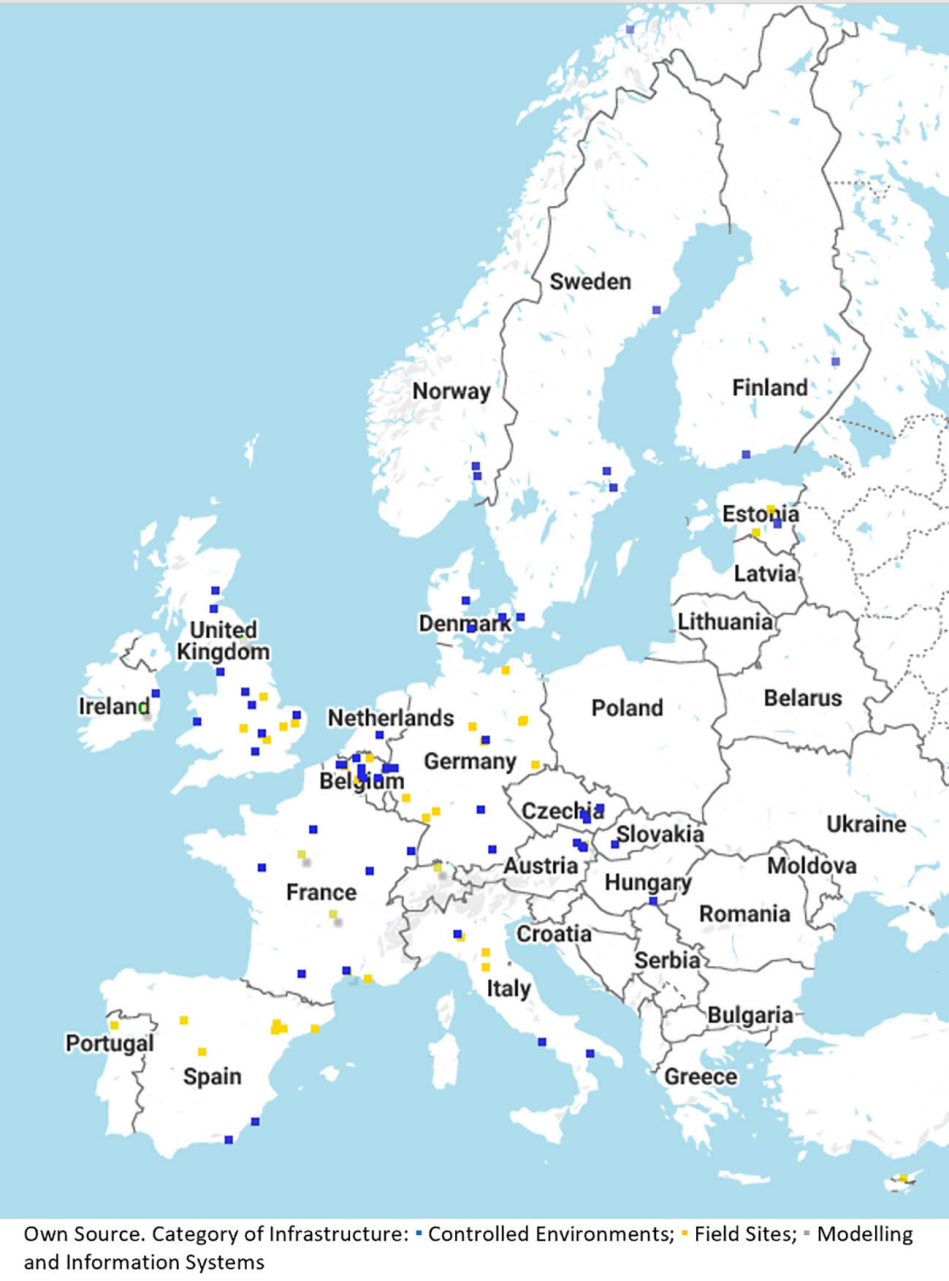
Overview of network for automated phenotyping technologies within research infrastructure.

In fiscal terms, this necessitates long-term investment in equipment and human resources (Mazzucato, 2016). In RIs for breeding and agricultural purposes, long-term experimentation is important (e.g. considering breeding cycles taking 10 years and more, Gerullis et al., 2021). Experimental set-ups need to go beyond the usual 3-year project term and limited field space to bring about useful and accurate long-term results. With the current set-up of phenotyping networks in Europe (see **Figure 2** for the Emphasis RI) it is possible to leverage multiple locations and installations distributed across Europe even though individual scientists may not have the same access to specialized installations at their home institutions.

There is a necessity to keep a good portion of scientific expertise within the RI as it needs maintenance and building up expertise for smooth workflows (ESFRI (European Strategy Forum on Research Infrastructures), 2020; Knowles et al., 2021). Long-term human resource development must be applied to scientists in the same way it is usually done in private businesses. While high-throughput phenotyping will need the same level of highly trained scientific staff, it will ease the shortage in person-power of technical staff for phenotyping large amounts of plant materials. Yet, technical knowledge on installations being run needs to be maintained over time as well and allowed to evolve further.

Individual scientists need to find an environment fostering collaboration across a wide range of disciplines and working cultures, who need to find new and transdisciplinary ways of solving research challenges (Brown et al., 2015). Transdisciplinary research needs disciplinary specialists *and* generalists who function as boundary actors between these different disciplines (Poteete et al., 2011). Hiring and maintaining the right set of people will determine success or failure of these infrastructures. Evaluation criteria for scientists working in research facilities connected to infrastructures determine the type of individuals joining different projects (Brown et al., 2015), research venues, and the success in using technological installations over longer time horizons. From climate change science we can learn that team science is key in solving complex challenges at hand and one can safely assume that sustainable agriculture is similar (Ledford, 2015; Cundill et al., 2019). Likewise, integration of social sciences is vital for tackling research challenges such as social system feedbacks (Viseu, 2015). For example, having a few social scientists that “speak plant” may help elicit unknown areas of knowledge between what breeders have been selecting for with “breeders eye” (Timmermann, 2009) – i.e. implicit knowledge on how breeding input traits translate into farm output traits in plants – and what pre-breeding scientists can see with their new sensors for enhanced vision. Such insights have potential to improve the effectiveness in implementing new breeding strategies, farming practices complementing newly bred plants, and extension services.

On an organizational level, polycentric governance of plant breeding requires RIs to build cooperative relationships amongst different actor groups to ensure effective research towards reaching mission goals (Lubell and Morrison, 2021). Scientists need to co-produce with farmers, breeders, agri-business, and citizens what sustainable traits in crops are and how they manifest in the food and fiber supply chain. Note though that each of these groups needs separate consideration in transdisciplinary approaches (Max-Neef, 2005).

Integrating farmers and food processors into the trait development process may also be of advantage when fruit attributes like thicker skins can enhance shelf life, for example in horticultural breeding. This could be done in a business-to-business context. An option would be to actively build platforms for public and private research cooperation by supporting start-up incubators with a focus on plant breeding (Shkolnykova, 2021) or to target participation of food processors in trait development as in the EMPHASIS RI context withthe Agroserv project (https://emphasis.plant-phenotyping.eu/european-infrastructures/cluster-projects/agroserv). Another option is integrating crop producers in participatory breeding processes (see Ceccarelli and Grando, 2020 for an overview) or in an extension service context, where extension employees survey the needs of the producers to allow plant breeders to make use of the knowledge on demanded traits.

Integrating non-scientific actor groups early on spells-out issues usually leading to unforeseen transition risks and lack of adoption (Mazzucato and McPherson, 2019). An example is the considerable societal resistance in Europe towards GMs and their ban from most agricultural use thereafter (Directive 2001/18/EC). Incorporating a dialogue with stakeholders and the public may lower transition risks and can be used as an opportunity for collective learning and diffusing innovations in public interest. Using and including governmental organizations already in place, such as agricultural extension services should be tried early on in development and testing processes, as it provides a notion of feasibility of traits in farm management practices.

How private businesses are integrated into a phenotyping network providing public services for research will greatly influence the effectiveness of delivering research insights. ‘Toxic actors’ can have detrimental effects on whole research venues and hamper their effectiveness in delivering research outcomes (Lubell and Morrison, 2021). Conflicts of interest may arise around data and material sharing, or specific methodological insights that constitute trade secrets. Mitigating these problems relies partially on a shift in mindset and ethics, towards more sharing attitudes and balancing incentive structures towards long-term goals over short-term revenues. A recent example within the EU RIs is the ENVRIplus project, which developed ethical guidelines for RIs (Capua et al., 2018) explicitly mentioning reciprocity amongst their guiding values. Including private actors may enhance testing capacities and promote insights if data is shared in a FAIR manner and symbiotic relationships are fostered (Mazzucato, 2016). Public value creation must be in focus of those taking care of research contracts over new projects for effective long-term risk and reward sharing (Mazzucato, 2016). Risk and reward sharing needs to be implemented such that they maintain an open innovation culture, which reinvests into further research. For example, in the RI Cluster project, CORBEL, governing guidelines for industry collaborations are provided to support this (Abuja et al., 2019).

Overall, the success of RIs will depend on how well its staff strategizes over knowledge, relationships, and decisions for implementation toward mission goals (Lubell and Morrison, 2021).

## 6 Conclusions

Mission-oriented governance for research is supposed to be implemented for plant breeding research to fulfill the SDGs and facilitate green growth. Improving crops through plant breeding will be vital for reaching the SDGs associated with agriculture. Crop breeding research shall bring about varieties enabling the necessary transformations to agricultural systems. High throughput technologies for phenotyping are meant to accelerate the plant breeding process and enhance breeders’ vision of breeding materials, leveraging innovation pathways. Yet, against the backdrop of complex agricultural systems and polycentric research venues, and agricultural governance, the question remains how to reach these ambitious goals.

We propose a governance heuristic illustrating how mission-oriented governance can work for plant breeding research. We show the current state-of-the-art of phenotyping technologies and draw, based on historic examples from plant breeding, implications for their introduction to individual breeding programs and RIs.

Our core result is that plant breeding is not only about the interaction of genetics (G), environment (E), and farm management practices (M), but that activities at collective level (S) are crucial for the sustainability performance at lower levels of the system. Hence, we propose GxExMxS as a guiding rule of thumb for future governance of plant breeding. This heuristic needs to be interpreted in specific context of application, e.g. when a funder wants to decide if a research project for plant breeding may be justified they may ask how novel plant traits lead to results on a higher level in the social-ecological system.

Additionally, we want to caution that novel phenotyping technologies alone will not bring about sustainable agricultural systems. Integrating robotics, sensors, and information systems meaningfully is necessary to elevate mental models of breeders, scientists, and other actors contributing to crop breeding. This implies a high heterogeneity in potential adoption of these technologies in breeding programs. Concurrently, RIs need to care how they institutionally navigate their role as facilitator and promoter of research to reach mission goals.

## Author contributions

MG is first author and wrote the first draft of the manuscript, conceptualized the paper. TH contributed to structure. SF, RP, LH and US edited sections of the manuscript. All authors contributed to the article and approved the submitted version.

## Footnotes

1 The same is also true for the even earlier domestication of animal species.

2 Usually termed as first generation GMOs, not to be confused with second generation GMOs who were altered for farming output traits like nutrient profiles of plants or third generation GMOs used for medical uses like insulin production.
